# Gleanings from Our Exchanges

**Published:** 1872-07-15

**Authors:** 


### Gleanings from Our Exchanges.

For the reduction of strangulated hernia without operation, Sir James Paget lays down the following general rules:

In cases, for instance, when the patient vomits fæcal matter and has peritonitis, or is in collapse, with a small, rapid pulse, hiccough, or other such extreme signs, there should be no attempt at reduction without operation.

When the coverings of the hernia are so inflamed as to make it probable that sloughing or suppuration has taken place beneath them, reduction should not be attempted without operation; and even when less inflamed, none but slight and brief efforts at reduction should be made.

The longer the signs of strangulation have existed the shorter should be the efforts at reduction, but the intensity of pain in recent or acute hernia should not deter one from making the attempt.

In a hernia which has been habitually irreducible and becomes strangulated, you should operate at once. It is a safe rule of practice that, after a warm bath and a few hours' rest in bed, a single attempt at reduction should be made; should this fail, chloroform or ether should be given, and then in some cases, but not in all, a second attempt made; this failing, the operation should be performed while the patient is still insensible.

The hot bath is useful in all cases that are not bad, unless in old and feeble persons; the patient should be simply soothed or relaxed in the bath, then wrapped in warm blankets, put into bed lying on his side or his back, with his knees drawn up, or with his pelvis a little raised, and then after an hour or two of complete rest to attempt the reduction. The employment of rest and the bath is helped by opium when the hernia is painful. In the old, and others who may have had inactive bowels long before the strangulation, an enema of a large quantity of liquid should be used. Purgatives should not be used if there are marked symptoms of strangulation.

After the warm bath and rest have been tried, you may give chloroform or some other anæsthetic. In making the attempt at reduction you must be gentle and self-restraining, mindful of the delicacy of some of the structures you are handling, and that you may do them much more harm than would come of the operation which you are trying to arrest. These cautions are the more necessary because when the patient is under chloroform, you have nothing but your own sense and senses to tell you how far you may go without doing harm. Chloroform is most useful in the herniæ of which the difficulty of reduction is chiefly due to muscular resistance, in the recent, or in the recently much enlarged; in the inguinal more than in the femoral; and in these more than in the umbilical; in the painful more than in the painless. In herniæ that have only recently come down, and are intensely painful, it is right to use chloroform or ether without waiting for the influence of the warm bath, but more commonly, if there be danger in waiting three or four hours, it is because strangulation is so far advanced that the operation ought to be done without any previous attempts at reduction.

After the warm bath, rest, and chloroform have been tried, and the reduction is not accomplished, and strangulation exists, you should operate while the patient is still under the influence of chloroform; but if strangulation is not present you may wait, but must watch patiently, for the hernia is likely soon to become strangulated. While waiting, ice or warm dressings, enemata, aperients or opiates may be used. Tobacco and curious postures, and shaking the legs up and the head down, and the cupping glasses are more dangerous than the operation which they are intended to avert. For doubtful or partial reduction there is one practical rule—operate if the symptoms of strangulation are not relieved. In cases in which reduction seems complete but the symptoms of strangulation are still present, operate, if you can feel a lump at or near the hernial ring.

Old age and disease may add to the risk of an operation for strangulated hernia, but they must be accepted. A patient must not be allowed to die with a strangulated hernia, if by any means whatever the strangulation can be relieved, and you must not be averted from the operation by the number of deaths that follow it. The deaths after the operation may be fifty per cent., but the deaths due to the operation are not more than two or three per cent.

The remaining lectures on this subject by Sir James Paget are devoted to a description of his several operations for the relief of strangulated hernia, which our space will not permit us to give to our readers.—*The Doctor.*

---

Milk as a Diet during Lactation. By R. P. Harris, M.D., Pennsylvania.—From a series of trials which I have very successfully made, I have become convinced of the great value of milk as a food for delicate mothers who desire to nurse their own children. By the term "delicate," I do not mean those actually diseased, or apparently inclined to tubercular or other serious organic affections, but a large class of American women in the higher walks of life, who fail as nursing mothers, either because their milk is too small in quantity, or deficient in nutritive elements. Such women are generally below their proper average in weight; have little, if any, color in their cheeks, and eat but a moderate amount of food. There may not be any deficiency in the development of their mammary glands, although their mammæ are usually smaller than they should be; but this is chiefly due to the absence of adipose deposit. All such subjects do not bear a milk diet well; and in such the plan must be abandoned, as the diet should not only agree with the mother, but be palatable so as not to diminish her appetite for her ordinary diet. She should be able to eat her three meals as usual, and consume the requisite amount of milk in addition. There are many women who have lost all their childhood's relish for milk, just as there are sometimes young children who do the same thing, and cannot be made even to try its efficiency. And there are others who are anxious for success, and do make the trial faithfully, but are reluctantly obliged to discontinue the diet in consequence, not of any disrelish, but of an inability to digest it.

Happily, there are also many who not only like the taste of milk, and can continue its use indefinitely, but who experience a wonderful degree of benefit from it, not only being able to nurse their infants, whom they would otherwise have to wet nurse or raise by hand, but greatly improved in health and strength, gaining flesh, increasing in appetite, and avoiding the ills resulting from the drain upon their system, so commonly experienced after a few months of lactation.—*Richmond and Louisville Medical Journal.*

---

Good Fortune of the Massachusetts General Hospital.—A few days ago the Treasurer of the Massachusetts General Hospital receipted for the large sum of $446,000, which was paid to him as the result of a provision of the will of the late John Redman. Mr. Redman died some twenty years since, leaving a will by which the hospital was made the residuary legatee of his estate upon the death of a son who had a life interest in it. The advance in both real and personal estate since that time has increased the value of the legacy from the $50,000, which the trustees of the institution once expected to receive, to nearly half a million.—*Boston Medical and Surgical Journal.*

Is Vaccine Lymph Curative as well as Preventive?—We find in the *Lancet* for May 25th ult., a communication which appears to open to view a new field in the discussion of the beneficence of vaccination, and which indicates that, under certain conditions, the vaccine lymph may be curative as well as prophylactic of variola. In this communication, Dr. Furley, of Edinburgh, describes his method of operating, and cites cases in his own experience to show that small-pox may actually be aborted by vaccination *after the variolous eruption has appeared.* Success, however, appears to depend greatly on the amount of virus exhibited and on the manner of its introduction to the blood. Dr. Furley thinks that he found that the ordinary method of vaccination, by scarifying the arm, is inoperative to modify the disease in adults, although it will sometimes be effectual in children. He accordingly used the subcutaneous method, at first injecting the lymph with the ordinary hypodermic syringe; but, subsequently, on finding that this process occasionally failed, by means of a hollow needle with a bore sufficiently large to admit a tube of virus. By this latter method the point of the needle is introduced "beneath the skin" and the lymph is blown, he says, from the tube "directly into the blood."

Three cases are reported in which the treatment by vaccination gave good results; the cases being selected indiscriminately from about sixty similar ones. In one, a baby aged one month, unvaccinated, the papular eruption was over the face, hands and legs. Ordinary vaccination was practiced, and in twenty-four hours the eruption had disappeared, except two papules on the face. On the third day these two papules had disappeared and a crop of four-and-twenty developed, chiefly on the head. In three days these had gone. The vaccination itself showed no signs of taking till the tenth day, and was matured on the fifteenth.

In a second case, a girl aged 13 years, vaccinated in infancy, appeared with the papular eruption on the hands and forearms. Two tubes of lymph were injected into the forearm. After three days, there was "nothing to be seen but the inflamed areola" at the point of injection.

Case three was an adult, unvaccinated. Two tubes of lymph injected on the second day of the variolous eruption modified the disease so that a probable confluent form became discrete, and desiccated on the ninth day. There was no areola at the point of injection.

The treatment by vaccine lymph injection is more effectual, according to Dr. Furley, the earlier it is practiced, and it is more successful in the youthful than in the adult period of life. Five unfavorable cases out of sixty are reported, in three of which the prognosis was bad from the beginning and in two death was unlooked for.

We regret that Dr. Furley does not describe more particularly the method of his injection of lymph—to what depth the needle is introduced into the tissues, and whether the lymph is all injected into a single puncture. It would also be interesting, in view of certain recent experiments in hypodermic vaccination, to know more in detail the appearances presented by the vaccination itself, *as modified by the method resorted to*, or *by the variola*, or by other unusual conditions present in such cases.—*Boston Medical and Surgical Journal.*

---

Subcutaneous Injection of Morphia and Chloroform.—In *El Progreso Medico*, April 1, we see it mentioned that Dr. Nussbaum has, from a desire to lessen the dangers of chloroform by diminishing the dose necessary to obtain anæthesia, made use of subcutaneous injections of morphia simultaneously with chloroform inhalation. The combination of the two substances, it seems, has been studied by Claude Bernard with good results, and has been applied to surgery, and also in obstetrics. From experiments made by MM. Labbe and Goujon, of which an account was given to the Academy of Sciences (*Gaz. Med. de Paris*), it results that by making an injection of small doses of morphia before giving chloroform, we obtain a far longer sustained anæsthesia, with very little chloroform; and it is certain that the dangers of this medicament are in direct relation with the quantity breathed by a patient. This is a great step, says the *Progreso Medico*, since great risk is spared to the patient, whilst at the same time the local and general sensibility of the patient is lessened.—*The Doctor.*

---

Electro-Therapeutic Cure of Catalepsy.—Dr. Holst, of Riga (*Dorpat Med. Z.*, 1872), mentions a case of a girl, aged seventeen, who was affected with cataleptic symptoms so bad as to be confined to bed, perfectly rigid, took no food, and was unconscious for a fortnight. By means of passing the constant current, 30 elements, through the vertebræ of the neck and back, she gradually recovered. The author considers that catalepsy is a spinal reflex excitability.—*Ibid.*

Hypodermical Injection in Obstetrical Operations.—In the *Independente* we find a quotation from the *Lyon Medical* as follows: "We know how difficult the complete evacuation of the amniotic liquid and the spasmodic contraction of the uterus render the execution of version. To favor the manœuver the use of chloroform is recommended; but if some practitioners have found this of use, others have obtained no advantage from it. Dr. Melvin Rohrer has added a method which has also been most successful in the practice of Dr. Brown, of Vienna, that is, the subcutaneous injection of morphine. In the case of a woman aged thirty, robust and of good health, and who had already given birth to three children without the aid of the obstetrician, three hours had passed since the waters burst, the belly was tense and sensitive to pressure, the pains returned with short intervals, whilst the exploration of the vagina was painful. An arm of the fœtus, violet and swollen, was in the vagina, and the corresponding shoulder was deeply wedged into the cavity of the pelvis. The patient was prostrated from pain. One-sixth of a grain of morphia was injected at the level of the linea alba at an equal distance between the umbilicus and pubes. Five minutes afterward the spasmodic contraction of the uterus was sensibly weaker, the intervals between the pains longer, and in twenty minutes there was complete calm; the uterus was soft, relaxed, and the shoulder became movable in the pelvis. Version was easily performed in a short time, and the fœtus was extracted without uterine contraction. Some slight frictions on the abdomen aided in the extraction of the placenta aud the case did well."—*The Doctor.*

---

A New Method of Arresting Epistaxis.—Dr. F. Marin, of Geneva (*Journal de Med. et de Chir. Prat.*, May, 1872), has discovered a new and simple method of arresting hæmorrhage from the nasal cavity. It consists in applying pressure to the facial artery at a point immediately beneath the ala of the nose, where the vessel can be pressed against the maxillary bone. In epistaxis the hæmorrhage is usually confined to the anterior third of one of the nasal fossæ, and as pressure upon the facial artery causes a diminution in the flow of blood to this cavity, the hæmorrhage is arrested almost immediately, and this proceeding is therefore recommended as preferable to that of plugging the posterior nares by the aid of Belloc's sound, in attempting which the surgeon is generally pretty thoroughly smeared with blood, and is not unfrequently bitten. Dr. Marin has had occasion to give numerous trials to this method suggested by him, and has generally found it effective. In two instances where it failed, plugging the posterior nares was attended with like result.—*Boston Medical and Surgical Journal.*

---

Respiratory Murmurs.—At a recent meeting of the New York Academy of Medicine, Dr. James R. Leaming read a paper on "Respiratory Murmurs," intended to show its composite character. Referring to the anatomy of the tissue of the lungs and bronchial system, he calls particulur attention to the circulations in the bronchial system; the bronchial arteries are attended by venæ comites only so far as the bronchial tubes extend, and not into the true pulmonary structure, consequently the blood which goes to nourish this structure does not return to the heart as venous blood, but is aerated in the air vesicles and returned by the pulmonary vein as arterial blood to the right side of the heart. Occupation of the air-sacs by tubercle interferes with the circulation of the nutrient artery, and blood is thrown back upon the bronchial artery, resulting in bronchoragia—a conservative act, for, like the application of leeches, it sets the absorbents actively at work to remove the cause. In this way cases of early phthisis are self-cured, or, at all events, ameliorated, and the physician is guided in his treatment. After some further comments on this manner of circulation the writer goes on to speak of the residual air in the lungs, and that it has no more motion than the water in the bottom of the ocean—the air in the air-cells is purified by the laws of the diffusion of gases. Hence, any theory of vesicular murmur or crepitant rale which ignores the existence of this residual air must of necessity be incompetent. The respiratory murmur consists of two sounds: the first, the motion of the tidal air in the bronchæ; the second, due to the contraction and dilatation of the muscular (?) fibers of the lung substances; the first is intermittent, the second continuous, and may be heard best when the breath is held. This, the true respiratory murmur, only begins to be developed after eight years of age, becomes recognizable at twelve, and is only fully developed at maturity. *The Broncho-Respiratory Murmur.*—The sound produced by the tidal air may be heard by forcing the respiration, thereby increasing the velocity of the air in the bronchial tubes and the loudness of the sound. It is heard most perfectly, however, in children before the true respiratory murmur begins.—*The Doctor.*

Treatment of Articular Rheumatism by Ice.—In the *Gaz. Med. de Strasbourg*, Feb., 1872, we notice that Dr. Esmarch published in the *Langenbeck's Archiv* about 1861, some observations on this subject. In the past winter there presented themselves in the ambulances four cases of acute rheumatism, which were treated by means of ice alone. In the first there was great pain in the joints of the feet; during the day the affection extended to the knee-joints, where ice was locally applied. On the morrow, the superior extremities were also affected with pain, the pulsations of the heart were weak, and the first sound accompanied by a murmur. All the painful articulations were covered with bladders containing ice. On the fourth day the pain had almost disappeared; on the fifth the pulsations of the heart were normal; on the sixth there were no longer any local symptoms; on the seventh the fever had ceased.

Two other cases were similarly followed by equally favorable results in the space of four days. In a fourth case ice was not used until the fifth day, but in four days after this the pain had disappeared with all other symptoms. Before the ice was applied the temperature increased gradually, but after its application it gradually became lowered. The author says he would not hesitate to use ice even in cases of gout; and as to the metastasis of rheumatism to the brain, he considers this to be the effect of the temperature. At Kiel there was received into the hospital a petient with rheumatic delirium, and with a tamperature of 43° C. He was immersed in water, and a cure took place. The application of ice should be continued until all symptoms disappear. The author is convinced that when the prejudice against the use of ice is overcome, it will be used often in acute joint affections. Ice-bladders should be used, and not cold compresses, as Priestnitz did.—*The Doctor.*

New York State Inebriate Asylum, Binghamton, N. Y.—The annual report of the Superintendent and Physician, for the year 1871, has come to hand. Number of patients received since the Asylum opened, May 1, 1867, 1,017. Number of Patients in Asylum January 1, 1871, 71. Patients admitted since January 1, 1871, 244. Patients treated, 315, and 230 discharged. Present number of patients, 85. Of the 230 discharged, 184 were discharged with great hopes of a permanent reformation. Discharged unimproved, 46. Of 244 admitted during the past year, 226 used tobacco, 149 were married, and 95 were single men. Age of the oldest patient, 63; of the youngest, 19; average age, 36. Deaths, none. 147 were constant drinkers, and 97 periodical drinkers. 165 drank whiskey; 64 indulged in brandy, gin and wines; 15 used opium. New York City contributed 27 patients; Brooklyn and other parts of the State 90; Connecticut, 11; New Jersey, 10; Ohio, 10; Pennsylvania, 18; Canada, 12; and Massachussetts, 9. Of the occupations, 19 were book-keepers; 29 were clerks; farmers, 15; lawyers, 25; merchants, 45; manufacturers, 14; physicians, 6; 21, no occupation. Daniel G. Dodge, M.D., about two years ago was placed in charge as Superintendent and physician of the Asylum. He is ably assisted by Mr. Carroll Hyde, Secretary, a former patient of the Institution. Dr. Willard Parker is the popular *President;* Wm. C. Wey, M.D., *First Vice-President;* George Burr, M. D., *Second Vice-President.*

Treatment of Scabies by Storax, Balsam of Peru, and Carbolic Acid.—Dr. V. Rothmund, Sen., of Munich (*Aert. Intell. Bl.*, 1871), mentions a mixture of two ounces of storax, olive oil one ounce, as an excellent and cheap remedy in itch, only that its scent is not good. Balsam of Peru is an excellent remedy in scabies. It contains cinnamic acid and ammonia. It is doubtless the best of all local remedies in itch, because the insects die more rapidly in pure balsam of Peru; it easily enters the skin, and baths are not required beforehand. A rubbing-in of about 40 tr. of Peruvian balsam is all that is required; repeated four or five times in the twenty-four hours suffices for a cure. Nine grammes are sufficient for an adult to rub in, and no bath is previously required. Dr. Rothmund also recommends the carbolate of sodium in quantities of half an ounce in six ounces of water applied to the affected parts thrice daily. This effects a cure in two days and a half.—*The Doctor.*

Virginia State Board of Health.—The newly established State Board of Health of Virginia was organized at Richmond, May 18th, by the choice of Prof. S. L. Cabell, of the University of Virginia, as President, and of Dr. L. S. Joynes as Permanent Secretary. The Board consists of seven members, all physicians. Among the matters brought before the meeting for immediate action was the subject of vaccination and re-vaccination, with special reference to compulsory legislation. Virginia is the second State to follow the example of Massachusetts in the institution of a Board of Health.—*Boston Medical and Surgical Journal.*

LOCAL TREATMENT OF SMALL-POX.—A writer in the London *Lancet* recommends the local application of liq. calc. sulph. in this disease, and reports great success therewith. We doubt its efficacy, as well as that of any other local application, but at the same time we are willing that others should try his method of treatment. He reports as follows:

"A boy of some ten or eleven years of age, who on Sunday last first showed the papular rash *very thickly* spread over the face, and fairly abundant over the body, whose primary fever had been very violent, with wild delirium, had the lotion applied over the whole body. I saw him yesterday (Wednesday), and found him quite well; the papules remained papules, and proceeded no further in development, but showed minute crusts on the surface of each papule, and where several were clustered together, and would evidently have become confluent, they remained in *statu quo*, arrested and destroyed. I am quite confident that had the disease been allowed to have its course, a serious and anxious case would have resulted; but by aid of the liq. calc. sulph. he is now in good health, with good appetite, and anxious to be up and down stairs.

"The boy's father took the disease while nursing a friend, and when I saw him the papules were becoming vesicles. By the application of the liq. calc. sulph. they *immediately* became pustular and died away; but he tells me he has had no scabs, and, having seen the course of the disease in his friend, is astonished and delighted with the lotion, and, therefore when, upon recovery, his son and wife were attacked, he immediately applied the lotion in the papular stage, with the remarkable result already stated."—*Medical Herald.*

---

TO TEST GREEN PAPER FOR ARSENIC.—We have been asked for a simple method of doing this. The tests for arsenic, strictly so called, are suited only to laboratory use, but since it is the arsenite of copper that is employed for the poisonous green colors, a test for *copper* is sufficient for ordinary purposes. Put a drop of aqua ammonia on the suspected paper, and if it changes the color to blue, you may be sure that copper is there, and almost as sure that arsenic is present also. There is not one chance in a hundred that a more critical examination would lead to a different conclusion. At any rate, we advise our readers not to use any paper on the walls of their houses, or for any other purpose, if this simple test makes its character suspicious.—*Boston Journal Chemistry.*

A MEDICAL CONGRESS, which is to be opened at Lyons on the 18th of September, is to be the medium of discussions upon the following subjects:—1. Epidemics of Small-pox. 2. Gun-shot Wounds. 3. Ambulances in Time of War. 4. Cattle-Plague and the Contagious Typhus of Cattle. 5. The Causes of the Decrease in the Population of France, and the means of arresting it. 6. The Treatment of Syphilis. 7. The Reorganization of Medical and Pharmaceutical Education in France. 8. The Means of Improving the Social Position of Medical Men.

---

DEATH FROM THE PRESENCE OF A PIECE OF NEEDLE IN THE HEART.—The *Cincinnati Lancet and Observer* states that at the post mortem examination of Miss Hoag, of Evanston, Ill., it was found that death was caused by the presence of a piece of needle in the heart. The needle was driven in by a sudden fall when she was about seven years old. The portion of needle extracted was about an inch and one-eighth long and of large size. The Editor remarks, "How life could have existed after it entered the heart, is a mystery even to medical men."

---

COMPARATIVE MORTALITY OF PHTHISIS IN DIFFERENT STATES.—Dr. Constantin Paul (*Rev. de Therap.*) mentions that in France the mortality from consumption is 10 per cent., whilst in Paris it is 13·4 per 100. If the statistical records of other countries are credible, in Rome it is 6 per 100; in Naples, 8; in Venice, 8; in Turin, 9; in Geneva, 9·7; in England, 11; in Belgium, 16; in Berlin, 17·5; in Vienna, 20; in Hamburg, 22; and in Boston, U. S., it is 23 per cent. In general, the mortality from phthisis is less in temperate climates.—*The Doctor.*

---

DR. GAUSTER, OF VIENNA, ON CHLORAL HYDRATE.—In the current number of the *Memorabilien*, Dr. Gauster mentions a case of tetanic spasms in a woman pregnant five months, which he succeeded in successfully treating by subcutaneous injection of morphia, and one drachm of chloral given in Liebreich's formula. In seven days of treatment the attacks of tetanus were arrested.—*Ibid.*

---

A PALPABLE HIT.—The *Boston Medical and Surgical Journal*, one of the very best of our exchanges, is responsible for the following thrust at the legal fraternity:

"The Criminal Code admits a new form of indictment in these latter days: Assault with intent to become insane."—*Medical Herald.*

Is Suicide a Sign of Insanity?—In the Superior Court of Baltimore, a verdict has been rendered against the Germania Life Insurance Company, of New York, for $2,000, the amount of a policy on the life of Lewis Fallman, who committed suicide in 1871. It was held the company was liable if the jury found the deceased had killed himself in a fit of insanity.

Such companies hold it *priori* evidence of insanity to commit suicide. Such a view is inconsistent with history and experience. Time was, when it was held an honorable thing to do, as witness the ancient Romans and Japanese. It depends on theoretical views of life and death whether it is or is not a rational and sensible act under many circumstances. These considerations have never been more forcibly put than in Hamlet's soliloquy, commencing, "To be, or not to be."—*Med. and Surg. Rep.*

The Lung Test.—M. Poucet showed, at a meeting of the Lyons *Societe des Sciences Medicales*, the lungs of a fœtus prematurely born. The child had cried, breathed, and lived an extra-uterine life of ten hours; but the lungs sank completely in water, as if no respiration had taken place. Other cases of the same kind have been related by other observers, showing the insecurity of the lung test in forensic medicine.—*Lancet.*

